# 

**Published:** 1862-09

**Authors:** 


### BOOKS AND PAMPHLETS RECEIVED.

*Communications of the Rhode Island Medical Society for the year* 1862. *Published by the Society.*

*The Retrospect of Practical Medicine and Surgery, being a half-yearly journal, containing a retrospective view of every discovery and practical improvement in the Medical Sciences. Edited by* W. BRAITHWAITE, M. D., *lecturer on Obstetric Medicine at the Leeds School of Medicine, etc., and* JAMES BRAITHWAITE, M. D. *New York: Published by* W. A. TOWNSEND.

This semi-annual publication is exceedingly valuable, containing gleanings of great practical importance in surgery, practical medicine and midwifery, besides a long catalogue of miscellaneous subjects. Physicians should not fail to buy and read this work.

*Annual Announcement of the Medical Department of the University of Buffalo, for the Session of* 1862–63. The regular term will commence on the first Wednesday in November, and continue sixteen weeks. Private instruction will be given to those students who may desire it during the term, or in the interval, upon—

*Chemistry*, or any other department of the Physical Sciences, by Prof. HADLEY.

*Physical Expectorations, Diseases of the Heart and Lungs*, by Prof. ROCHESTER.

*Physiology*, and upon *Microscopy in its application to the diagnosis of morbid growths, Urinary Deposites, &c., &c.*, by Prof. MASON.

*Annual Report of Saint Vincent's Hospital, corner of Eleventh Street and Seventh Avenue, New York, under the charge of the Sisters of Charity, for the year* 1861. *By* J. J. CONNOLLY, M. D., *Resident Physician and Surgeon. New York:* D. & J. SADLIER & COMPANY, 164 *William Street*, 1862.

*College of Physicians and Surgeons, New York; Medical Department of Columbia College. Fifty-fifth Annual Catalogue of the Officers and Students of the College. Session of* 1861–62.

*The London Lancet, a Journal of British and Foreign Medicine, Physiology, Surgery, Chemistry, Criticism, Literature, and News; edited by* THOMAS WAKLEY, *Surgeon, M. P. during eighteen years for the Metropolitan Borough of Finsborough, and Coroner for the County af Middlesex; sub-editors*: J. H. BENNET, M. D., T. WAKLEY, JR., M. R. C. S. E.

This foreign monthly reprint, comes regularly upon our table and always contains much that is new, interesting and valuable. It is a journal which we advise all physicians to read.

*Announcement.—Medical Department of Western Reserve College, Cleveland, Ohio.* The Annual Session will begin on the first Wednesday of November, and continue sixteen weeks. A course of Military Surgery will also be given.

